# “One Size Doesn’t Fit All”: Design Considerations for an Exercise Program to Improve Physical Function in Older Veterans with Serious Mental Illness

**DOI:** 10.3390/ijerph22020191

**Published:** 2025-01-29

**Authors:** Julia Browne, Whitney L. Mills, Courtney T. Lopez, Noah S. Philip, Katherine S. Hall, Alexander S. Young, Kate M. Guthrie, Wen-Chih Wu

**Affiliations:** 1 Center of Innovation on Transformative Health Systems Research to Improve Veteran Equity and Independence (THRIVE COIN), VA Providence Healthcare System, Providence, RI 02908, USAwen-chih.wu@va.gov (W.-C.W.); 2 VA Providence Healthcare System, Providence, RI 02908, USA; 3 Department of Psychiatry and Human Behavior, Warren Alpert Medical School of Brown University, Providence, RI 02903, USA; 4 Department of Health Services, Policy & Practice, Brown University, Providence, RI 02903, USA; 5 Center for Neurorestoration and Neurotechnology, VA Providence Healthcare System, Providence, RI 02908, USA; 6 Geriatric Research, Education and Clinical Center, Durham VA Health Care System, Durham, NC 27705, USA; 7 Center for the Study of Aging and Human Development, Duke University School of Medicine, Durham, NC 27710, USA; 8 Mental Illness Research, Education and Clinical Center, VA Greater Los Angeles, Los Angeles, CA 90073, USA; 9 Department of Psychiatry and Biobehavioral Sciences, University of California Los Angeles, Los Angeles, CA 90095, USA; 10 Medical Service, VA Providence Healthcare System, Providence, RI 02908, USA

**Keywords:** physical activity, intervention development, schizophrenia, bipolar disorder, qualitative, rapid analysis

## Abstract

Older adults with serious mental illness (SMI) (i.e., schizophrenia, schizoaffective disorder, bipolar disorder) have compromised physical function that adversely affects their quality of life. Exercise is an effective intervention to improve function in older persons; however, older people with SMI experience barriers to exercise engagement. This study sought to obtain feedback on an exercise program in development for older people with SMI that comprised home-based exercise delivery, individualized exercise prescription, and motivational health coaching calls. Individual interviews and focus groups were conducted with older Veterans with SMI (*n* = 3) and clinical staff serving this population (directors: *n* = 3; clinicians: *n* = 15, k = 3) to elicit feedback on the perceived feasibility and acceptability of the preliminary program and recommendations for modifications to the program. Rapid analysis was used to summarize transcripts of audio-recorded interviews and focus groups. Results indicated a strong perceived feasibility and acceptability of the preliminary intervention because of how the individualized exercise prescription component (i.e., exercise plan) would be personalized to the Veteran’s preferences and abilities. Clinical staff participants expressed concerns about how the lack of real-time supervision would negatively affect exercise completion. Participants recommended tailoring the home-based exercise delivery and motivational health coaching calls components to each Veteran’s unique context.

## 1. Introduction

People with serious mental illness (SMI: schizophrenia, schizoaffective disorder, bipolar disorder) are at major risk for loss of independence and early mortality [1,2,3]. Medical morbidity and reduced physical function are central contributors to these negative health outcomes, both of which worsen with age. In fact, people with SMI, and older adults in particular, have high rates of chronic medical issues (e.g., cardiovascular diseases) that often result in increased emergency and medical healthcare utilization [4,5,6,7,8]. Older adults with SMI also show significantly reduced physical function across endurance, strength, and mobility domains compared to both adults without SMI and age- and sex-based reference values [9]. Overall, physical function levels observed in middle-aged persons with SMI resemble those of people 20-to-30 years older than their chronological age, pointing to accelerated aging in this group [10]. Therefore, interventions that target the reduced physical function observed in older adults with SMI have tremendous potential to improve the quality of life for this population.

Exercise is an effective intervention to improve physical function in older adults [11,12,13,14] and in those with SMI [15,16,17]. Yet, engaging people with SMI, and especially older adults with SMI, in exercise is challenging [18,19]. This population experiences a number of barriers that impact their participation in exercise programs including environmental challenges (e.g., limited access to transportation and gym facilities), low motivation stemming from mental health symptoms, and compromised health status [19,20,21]. These difficulties are especially hard to overcome in facility-based exercise interventions that inherently require individuals to have both the motivation and capability (physical and logistical) to leave home and attend an in-person program. Therefore, alternative options to face-to-face programs may be warranted to adequately engage older people with SMI in exercise-based interventions.

Home-based exercise programs could improve engagement in older people with SMI because they eliminate the need for travel and gym access. These types of programs are safe and effective at improving physical function in older adults [22,23,24,25]. Individualized home-based exercise programs, in particular, are valuable because they allow for tailoring the exercise prescription to meet the needs and functional capacities of the older adult [26]. Additionally, certain models of home-based exercise for older adults include ongoing support and accountability from health coaches [27], which can be crucial for maintaining motivation and engagement. For example, home-based cardiac rehabilitation (HBCR), a 12-week intervention with individualized exercise prescription, includes weekly telephone-based health coaching to support motivation and adherence [28]. Evaluations of HBCR have highlighted high levels of engagement as well as improvements in physical function and quality of life for individuals navigating major medical issues (e.g., a recent heart attack) [29,30]. Taken together, a home-based exercise program that includes the HBCR components of individualized exercise prescription and health coaching could address the environmental, motivational, and medical barriers that prevent older adults with SMI from adequately engaging in facility-based exercise programs. Yet, to date there have not been any home-based exercise programs designed specifically to address the specific challenges faced by older adults with SMI.

**Figure 1:**
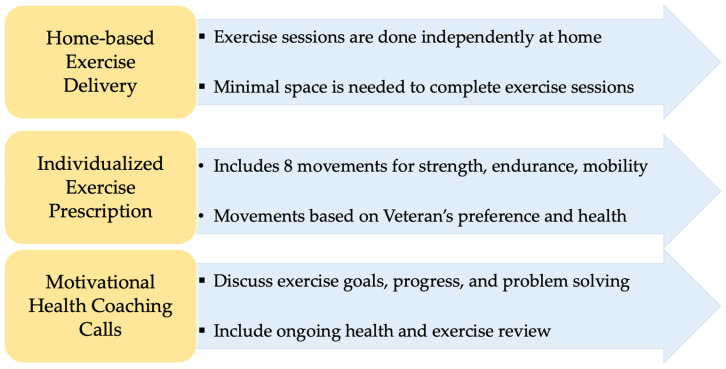
Preliminary exercise program components.

The purpose of this study was to obtain feedback from key informants, including older Veterans with SMI and clinical staff (directors and clinicians) serving the SMI population, on the design of an exercise program specifically for older Veterans with SMI. Guided by the HBCR model [28], the research team developed a preliminary 12-week program that comprised three components: (1) home-based exercise delivery (i.e., exercise sessions to be completed independently at home and that would require minimal space), (2) individualized exercise prescription (i.e., an exercise plan consisting of eight movements to be developed based on the individual’s functional capacity and preferences), and (3) motivational health coaching calls (i.e., phone calls to be delivered by a health coach to provide health monitoring and motivational support) (Figure 1). The study aims were to elicit (a) feedback on the perceived feasibility and acceptability of the preliminary program and (b) recommendations for modifications to the preliminary program.

## 2. Materials and Methods

This qualitative study was conducted at a Department of Veterans Affairs Healthcare System (VAHCS). Ethical approval was obtained from the VAHCS’s Institutional Review Board (IRB). Based on the IRB’s determination that this study met criteria for an exempt review category, written informed consent was not required. Study staff reviewed an approved study information sheet and obtained verbal agreement to participate from prospective participants before any study procedures took place.

### 2.1. Participants

Participants with SMI were eligible if they (a) were a Veteran enrolled at the local VAHCS, (b) were at least 50 years old, and (c) had a diagnosis of schizophrenia, schizoaffective disorder, and/or bipolar disorder in their medical chart.

Director and clinician participants were eligible if they provided clinical care in at least one of three pre-specified outpatient programs that serve Veterans with SMI at the local VAHCS.

### 2.2. Data Collection Tools

The research team developed a semi-structured interview guide that comprised eight queries designed to elicit (a) feedback on the perceived feasibility and acceptability of the preliminary program and (b) recommendations for modifications to the preliminary program. Parallel versions of the interview guide were created for Veteran and director/clinician participants that included minor wording changes to reflect the participant type (e.g., “Veterans like you” versus “Veterans with SMI that you serve”).

Queries in the feasibility and acceptability section were open-ended (e.g., “what did you think about the motivational health coaching calls?”) with several follow-up questions to probe for greater detail (e.g., “what do you like about this part?”). Queries in the recommendations section were specific and targeted to obtain input on the frequency and content of the program (e.g., “As you know, our program will last 12 weeks. How often do you think we should have the motivational health coaching calls?”). This section also included follow-up questions and an open-ended final query (“Do you have any additional thoughts or recommendations about our program?”) to probe for more detailed suggestions for the program design (Table 1).

### 2.3. Procedure

The first author (JB) conducted all interviews and focus groups remotely. She interviewed Veterans (via telephone through the Microsoft Teams platform) and clinic directors (via Microsoft Teams conferencing) individually. She interviewed clinicians in focus groups for each of the three pre-specified clinics (via Microsoft Teams conferencing). Of note, due to a technological issue, one focus group was interrupted prior to completion (after participants responded to question 5 in the interview guide: see Table 1) and was unable to resume as a group. The interviewer completed the remainder of interview questions (questions 6–8 in the interview guide: see Table 1) individually with participants following the interruption of the group. Individual interview data from focus group participants were compared with completed focus groups and data quality and credibility was deemed comparable. To align with the Veteran interviews that were conducted via telephone, clinical staff were encouraged to disable video capabilities during interviews and focus groups. Of note, two of the individual interviews were conducted with video capability enabled. All interviews and focus groups were recorded and automatically transcribed via Microsoft Teams in real time.

At the start of each interview and focus group, the first author provided participants with a verbal description of the preliminary program including its three main components (i.e., home-based exercise delivery, individualized exercise prescription, motivational health coaching calls). A visual depiction of the components was also shown to directors and clinicians via share-screen capabilities (Figure 1). The first author then conducted the interviews and focus groups following the semi-structured interview guide (Table 1). At the conclusion of each interview and focus group, participants completed a brief self-report form about their demographic information (and clinical background for director and clinician participants). Director and clinician participants completed the self-report form electronically via REDCap [31,32] and Veteran participants completed the self-report form verbally over the phone. Veteran participants received USD 25 for completing interviews.

### 2.4. Data Analysis

The data analysis team comprised two psychologists (JB, CTL) and a gerontologist (WLM). The team used rapid analysis, an efficient and effective approach to summarizing qualitative data that is particularly well suited for applied studies seeking action-oriented results within short timeframes [33,34]. Overall, the analytic procedure involved independent summarization of transcripts, weekly meetings to review independently developed summaries and input final combined summaries into a matrix, and visualization and interpretation of the completed matrix [35].

Both psychologists initially reviewed the templated summary table that was developed for analysis (Table 2) and determined that it adequately outlined the pre-specified domains. The templated summary table has two sections that align with the structure of the interview guide: (1) feedback on the perceived feasibility and acceptability of the preliminary program and (2) recommendations for modifications to the preliminary program. Within each section, the table has three columns: (1) a pre-specified domain derived from relevant interview guide questions, (2) interview guide questions that directly mapped onto the domain, and (3) space to summarize the domain. To assess the acceptability of the templated summary for use with individual interviews and focus group transcripts, both psychologists independently summarized one transcript of each type (one each week for two weeks) and then met weekly virtually to review the summarization process. They determined that the templated summary was thorough, clear, and acceptable for continued use with both types of data (i.e., individual interviews and focus groups) without changes.

Both psychologists then independently summarized all of the remaining transcripts over the course of four weeks. Each week during the independent summarization phase (including during the initial templated summary process review), both psychologists met virtually to review independent summaries, collaboratively determine an agreed-upon summary for each domain, and input the final summaries into the matrix. The third team member (WLM, gerontologist) was available to join weekly meetings to address any major discrepancies (note: there were no major discrepancies that arose from independent summarization). In the final stage of analysis, all three team members met twice virtually to review the final summaries for each domain and collaboratively identify key findings for each pre-specified domain within the two sections (i.e., feasibility/acceptability, recommendations).

## 3. Results

### 3.1. Participant Characteristics

Out of five Veterans that were screened, three enrolled and completed individual interviews. All three clinic directors that were screened completed individual interviews. Out of 25 clinicians that were screened, 15 enrolled and participated in one of three focus groups (*n* = 5, *n* = 8, *n* = 2). On average, individual interviews lasted 15.9 min (range: 10.9–21.8) and focus groups lasted 30.8 min (range: 27.0–34.6). Of note, the duration of the one focus group that was interrupted by a technological issue prior to completion and completed via individual interviews was not included in the average calculation.)

The overall sample of 21 participants (across Veterans, directors, and clinicians) included 10 women and 10 men (one individual did not disclose their gender). Most participants were white (*n* = 17) and not Hispanic or Latino (*n* = 18). Veteran participants were, on average, 56.3 years old (range: 55–57), none of whom were currently employed. Director and clinician participants were, on average, 43.2 years old (range: 29–62) and had professional backgrounds in psychology, medicine, social work, nursing, peer support, occupational therapy, and pharmacy. On average, director and clinician participants had 5.5 years of experience (range: 2–15) serving Veterans with SMI.

### 3.2. Perceived Feasibility and Acceptability of Preliminary Exercise Program

Overall, participants across all types (i.e., Veterans, directors, clinicians) expressed a high degree of perceived feasibility and acceptability of the preliminary exercise program. They described feeling that the program would be valuable for older Veterans with SMI to improve their health. They noted positive aspects of all three components of the program (i.e., home-based exercise delivery, individualized exercise prescription, motivational health coaching calls) while noting especially the high perceived feasibility and acceptability of the individualized exercise prescription. They felt that a program tailored to the Veteran’s preferences and abilities would be especially helpful as it could address the health issues and low motivation that older Veterans with SMI experience. The quotes below illustrate the enthusiasm for the program as a whole and especially for the individualized exercise prescription component:

“*I think it’s a great idea. I’m very excited that someone is wanting to help Veterans with SMI work on their physical health.*”

—Director

“*The individualized piece I think is really what’s gonna make this a lot more helpful and useful to each individual person [because it is] based on their individual contexts.*”

—Clinician

“*I think [the individualized exercise prescription is] good in my case, … I have sciatica … some people have arthritis, some people have this and that … weak knees, weak back, weak shoulder. So yeah, you could … incorporate what their medical needs are …*”

—Veteran

As opposed to Veteran participants who provided largely positive views of the program, clinician and director participants described some aspects of the program and its components that could negatively impact the feasibility, acceptability, and ultimate impact. The main concern from clinician and director participants was about how the lack of real-time supervision of exercise sessions could reduce motivation and participation. The quotes below illustrate this concern:

“*… I just would be concerned that who would be monitoring them during the exercises, thinking of some of my patients, they probably just wouldn’t do the exercises.*”

—Clinician

“*I think any time we’re asking Veterans to do things independently at home there [are] the usual challenges that come up in terms of … follow-through and … motivation …. It’s hard to get anybody to do things independently at home.*”

—Director

“*… I think compliance is gonna be an issue. I don’t think they’re gonna be able to do these exercises on their own.*”

—Clinician

#### 3.2.1. Perceived Feasibility and Acceptability of the Home-Based Exercise Delivery Component

Overall, participants across all types described that most older Veterans with SMI would be willing to exercise at home. Positive aspects of the home-based exercise delivery component were provided across all nine interviews/focus groups. Specifically, participants found this aspect beneficial to increase access to an exercise program for this population because it removes travel barriers that commonly affect this group’s ability to engage in face-to-face interventions. Further, they noted that Veterans with SMI are more comfortable exercising at home because it reduces the emotional barriers to engagement such as anxiety about going to the gym and/or shame around exercise abilities. The quotes below illustrate several benefits of home-based exercise delivery for this population:

“*[It would] be easier for me. … I [live far] from the [healthcare system]. Be tough to get to the [healthcare system for] exercising.*”

—Veteran

“*… [Older Veterans with SMI] may have … anxiety or they may not have a car or they may rely on … the bus to take them … so it’s imperative that [exercise] can be done … easily in in the confines of their home.*”

—Director

Participants in six out of nine interviews/focus groups (i.e., 1/3 Veterans, 2/3 directors, 3/3 clinician focus groups) described potential drawbacks and challenges of home-based exercise delivery. Participants expressed concern that exercising at home would be challenging for Veterans without adequate space in their homes. They also described concerns that motivation may be lower when exercising at home and that staff would have difficulty monitoring adherence and proper form in a home-based program. Further, they shared concerns that exercising at home does not promote socialization or community engagement, which could result in social isolation. The quotes below illustrate some of these concerns:

“*… I’m saying some people might, like, be lazy at home or … they might be doing [the exercise program] for socialization. … I think a handful may wanna come in [to an in-person program] to make friends, to meet people, to feel like they’re at a gym …*”

—Veteran

“*Can we truly monitor if the Veterans are doing the exercises properly if we can’t see [the Veterans]?*”

—Clinician

#### 3.2.2. Perceived Feasibility and Acceptability of the Individualized Exercise Prescription Component

Overall, participants across all types described that most older Veterans with SMI would be interested and willing to use an individualized exercise plan. Positive aspects about the individualized exercise prescription component were provided across all nine interviews/focus groups. Participants appreciated the ability to customize the exercise plan to meet the preferences, goals, and abilities of older Veterans with SMI. Further, they described how tailoring the exercise plan would foster greater motivation and participation in exercise among older Veterans with SMI. The quotes below illustrate the importance of individualizing the exercise plan:

“*So the fact that we could make [the exercise program] individualized and make that the big selling point could be helpful for folks because they’re getting that one-on-one attention and knowing that the movements recommended are specifically for their body rather than generalized.*”

—Clinician

“*Now I’m very overweight so I can’t do a lot of physically strenuous activities either, you know, so tailoring … to the person’s … health condition, to their physical body makeup and their age [is important]. So one size doesn’t fit all.*”

—Veteran

Participants in two out of nine interviews/focus groups (i.e., 0/3 Veterans, 1/3 directors, 1/3 clinician focus groups) described potential drawbacks and challenges of the individualized exercise prescription component. Participants shared concerns that older Veterans with SMI could feel overwhelmed by too many exercises on their plan and specifically with remembering how to correctly perform the exercises. The quote below illustrates these concerns:

“*I think … with some of the … maybe memory challenges that [older Veterans with SMI] have or … certain number of exercises … might be difficult for them to remember how to do … or to do them with good form, that sort of thing.*”

—Clinician

#### 3.2.3. Perceived Feasibility and Acceptability of the Motivational Health Coaching Calls Component

Overall, participants across all types described that most older Veterans with SMI would be willing to participate in the motivational health coaching calls. Positive aspects about this component were provided across all nine interviews/focus groups. Participants expressed that the calls would be important to increase and sustain motivation and engagement in exercise. Participants viewed the calls as opportunities for coaches to offer prompts/reminders to exercise, to review goals and problem solve challenges, to provide social support, and to create a sense of accountability. The quotes below illustrate the value of motivational health coaching calls for this population:

“*I think [motivational health coaching calls will] be really helpful to increase the compliance with the exercises. I think our Veterans benefit from prompts and reminders, and then also just motivational enhancement work.*”

—Director

“*I think [motivational health coaching calls are] necessary and the structure is good and it’s … support … it’s important to let [Veterans] know they are not alone.*”

—Veteran

Participants in six out of nine interviews/focus groups (i.e., 1/3 Veterans, 2/3 directors, 3/3 clinician focus groups) described potential drawbacks and challenges of the motivational health coaching calls. Participants noted that some older Veterans with SMI may not be interested in receiving phone calls, may be difficult to reach by phone, and may find ongoing calls difficult to manage with other commitments. The quote below illustrates one of these potential challenges:

“*You know, I got other appointments and calls and so [motivational health coaching calls] could be burdensome.*”

—Veteran

### 3.3. Recommendations for Changes to the Preliminary Exercise Program

Participants offered several specific recommendations for changes to the preliminary exercise program to enhance its feasibility and acceptability. Overall, participants across all types emphasized the importance of adding more personalization to the program to increase motivation and engagement. For example, they suggested collaboratively developing a schedule with the Veteran that would encourage them to identify the optimal days and times to complete their exercise sessions. They also recommended customizing the frequency of exercise sessions and motivational health coaching calls as well as the quantity of exercises on the exercise plan to the Veteran’s preferences, goals, and abilities. In addition, they recommended tailoring the content of coaching calls to meet the needs of the Veteran. The quotes below illustrate the importance of personalizing the exercise program to this population:

“*… Depending on the situation—if [Veterans] are making progress, maybe taper off [the number of motivational health coaching calls]. If [Veterans] are not making progress, up [the number of motivational health coaching calls] a little bit.*”

—Veteran

“*Power of choices get you a very long way with Veterans.*”

—Clinician

## 4. Discussion

This qualitative study elicited feedback from key informants (i.e., older Veterans with SMI and clinical staff serving Veterans with SMI) on the preliminary design of an exercise program for older Veterans with SMI. Participants expressed enthusiasm for the program and all three of its components including home-based exercise delivery, individualized exercise prescription, and motivational health coaching calls. Veteran and clinical staff participants perceived a high feasibility and acceptability of the program largely because of how the individualized exercise prescription is tailored to the Veteran’s preferences and abilities. Clinical staff, but not Veteran participants, reported concerns about how the lack of real-time supervision may not be sufficient to overcome motivational challenges in this population. Participants across all types recommended further incorporating personalization into other aspects of the program to better enhance motivation and engagement. Overall, results from this study highlight the importance of personalization when developing an exercise program for older adults with SMI.

Participants across all types strongly valued the individualized exercise prescription component of the program as it allows for the development of an exercise plan that is tailored to the preferences and physical capabilities of the older Veterans with SMI. Research has shown that, compared to younger Veterans with SMI, older Veterans with SMI are more likely to endorse specific physical health issues as barriers to exercise [36], emphasizing the potential value of considering medical status in exercise prescription. Further, a study of 114 persons with SMI demonstrated that exercise preferences varied based on certain characteristics including age and gender, further highlighting the need to consider an individual’s context when developing an exercise plan. The benefits of personalizing exercise prescription can also lead to improved self-efficacy and motivation, which, in turn, can promote better physical activity engagement [37]. Increasing motivation is especially important for people with SMI as it is often reduced in this population and considered a contributor to their low participation in exercise [38,39,40]. Taken together, individualized exercise prescription should be a central component of a program designed for older adults with SMI.

Participants across all types expressed positive views of home-based exercise delivery for older Veterans with SMI because it allows for greater accessibility and convenience. Removing travel and environmental (e.g., gym access) barriers are especially important for individuals with SMI given the high rates of dropout from face-to-face exercise interventions observed in this population [18,19]. Previous studies in young and middle-aged people with SMI have shown improved engagement in remotely delivered exercise and lifestyle programs compared to face-to-face interventions [41,42]. Yet, clinical staff participants noted concerns that motivation may be lower in a home-based program, particularly one without real-time supervision (i.e., monitoring/supervision during the exercise sessions) and, as such, could reduce engagement. Veteran participants, however, did not report these same concerns. Supervision during exercise has been associated with better engagement in people with SMI; however, few studies included older adult samples [19]. A recent meta-analysis of 34 studies in older adults (without SMI) found similar attendance rates for supervised versus unsupervised exercise programs [43], possibly suggesting that an unsupervised format may be adequate for the older adult population. Moreover, including real-time supervision could limit some of the program’s personalization (e.g., because exercise sessions would require specific scheduling around supervisor availability) [28]. Based on these findings, a home-based exercise program could improve engagement in older people with SMI. Given that clinical staff, but not Veterans, expressed concerns about the lack of real-time supervision, it may be most appropriate to provide periodic but optional opportunities for Veterans to receive this extra support either in person or through digital modalities.

Participants across all types viewed the motivational health coaching calls as important for providing the support, prompting, and motivational enhancement needed to engage older Veterans with SMI in exercise. Clinical staff participants shared that it can be difficult to reach Veterans with SMI by phone and clinical staff and Veteran participants described that it is possible that Veterans may become overly burdened by participating in the calls along with their other demands. Telephone-based health coaching has been effectively integrated into lifestyle programs for people with SMI [42,44] and can be more flexibly delivered than video-based or face-to-face support. As such, telephone-based health coaching is likely to be a valuable component of a home-based exercise program for older adults with SMI. Yet, assessing how the frequency and timing of calls align with a Veteran’s availability will be necessary to minimize the burden and enhance willingness to participate.

Participants across all types recommended further personalization of the exercise program to enhance motivation and engagement. Specifically, participants recommended integrating personalization into home-based exercise delivery and motivational health coaching call components to ensure that all aspects of the program are sufficiently tailored to the older Veterans with SMI. For example, encouraging the older Veterans with SMI to identify the most suitable day, time, and location for them to complete their exercise sessions can increase their autonomy and motivation. Additionally, tailoring the content of motivational health coaching calls to address specific challenges faced by each older Veteran with SMI can increase their self-efficacy to engage in exercise. These recommendations align with work that underscores the importance of supporting autonomy and increasing motivation in people with SMI to support exercise engagement [45,46]. Therefore, exercise interventions for older people with SMI should incorporate personalization into as many aspects of the program as possible to maximize motivation and participation.

This study had limitations that should be considered when interpreting the results. First, all participants were from one VAHCS and were primarily white and not Hispanic or Latino. Second, as this study was not primarily aimed at identifying differences in responses across types of participants, there was a small number of Veteran participants compared to clinical staff participants. As such, given the limited number of Veteran participants in the current study, Veteran data should be seen as preliminary in nature. Third, the extent of participants’ personal and/or professional experience with exercise was not measured, which could have impacted their responses. Future studies should include a larger number of Veteran participants from diverse racial, gender, and cultural backgrounds to enhance the generalizability of the study findings. Despite these limitations, this study offers valuable perspectives about an exercise program specifically designed for older Veterans with SMI. Particularly given the high prevalence of SMI among older Veterans (~11% with schizophrenia, ~4% with bipolar disorder) [47] and the reduced physical function capacities observed in this population [9], future intervention research is needed to develop an effective personalized home-based exercise program for this population. Future work should also evaluate potential mechanisms of change, particularly improved self-efficacy and motivation, to maximize the impact of the intervention.

## 5. Conclusions

This qualitative study examined feedback on a preliminary exercise program for older Veterans with SMI that included home-based exercise delivery, individualized exercise prescription, and motivational health coaching calls. Results from the rapid analysis of interviews and focus groups of older Veterans with SMI and clinical staff serving this population demonstrated high feasibility and acceptability of the overall program, particularly the individualized exercise prescription component. Participants recommended further tailoring the home-based exercise delivery and motivational health coaching call components to the preferences and abilities of the older Veterans with SMI. Overall, this study underscores the potential of personalization when designing a feasible, acceptable, and effective exercise program for older adults with SMI.

## Figures and Tables

**Table 1:** Interview guide initial queries and follow-up questions.

Interview Guide
Feasibility and Acceptability: Overall, what do you think about the exercise program? Follow-up 1a: Do you think (Veterans like you/Veterans with SMI that you serve) would participate in our program? Why or why not? What did you think about the home-based delivery (e.g., exercising at home)? Follow-up 2a: What do you like about this part? Why? Follow-up 2b: What don’t you like about this part? Why? Follow-up 2c: Do you think (Veterans like you/Veterans with SMI that you serve) would like this part? Would they be willing to exercise at home? Why or why not? What did you think about the individualized exercise prescription (e.g., having a plan of 8 exercises created for you)? Follow-up 3a: What do you like about this part? Why? Follow-up 3b: What don’t you like about this part? Why? Follow-up 3c: Do you think (Veterans like you/Veterans with SMI that you serve) would like this part? Would they be willing to use the plan? Why or why not? What did you think about the motivational health coaching calls (e.g., phone calls from our team to check-in and set goals)? Follow-up 4a: What do you like about this part? Why? Follow-up 4b: What don’t you like about this part? Why? Follow-up 4c: Do you think (Veterans like you/Veterans with SMI that you serve) would like this part? Would they be willing to participate in the calls? Why or why not? Recommendations: 5. How many times/week do you think (Veterans like you/Veterans with SMI that you serve) would exercise at home using our plan? Follow-up 5a: Is there anything you would recommend we do to help Veterans stick to their plan? 6. What do you think about the plan having eight exercises? Follow-up 6a: Would you suggest a different amount? Why or why not? 7. As you know, our program will last 12 weeks. How often do you think we should have the motivational health coaching calls? Follow-up 7a: Is there any specific information you think Veterans would want to receive during the motivational health coaching calls? 8. Do you have any additional thoughts or recommendations about our program?

Note. SMI = serious mental illness.

**Table 2:** Templated summary table.

Domain	Corresponding Interview Guide Question(s)	Key Summary *
Feasibility and Acceptability:		
Feasibility and acceptability of program	1, 1a	
Positives about home-based delivery	2, 2a	
Negatives about home-based delivery	2, 2b	
Feasibility and acceptability of home-based delivery	2c	
Positives about individualized exercise prescription	3, 3a	
Negatives about individualized exercise prescription	3, 3b	
Feasibility and acceptability of individualized exercise prescription	3c	
Positives about motivational health coaching calls	4, 4a	
Negatives about motivational health coaching calls	4, 4b	
Feasibility and acceptability of motivational health coaching calls	4c	
Recommendations:		
Frequency of exercise sessions	5	
Strategies to increase exercise follow-through	5a	
Quantity of exercises on exercise plan	6, 6a	
Frequency of motivational health coaching calls	7	
Recommended content for coaching calls	7a	
Other feedback and recommendations	8	

* Information relevant to the key summary may appear anywhere in the transcript (not just in response to the specified interview guide question(s)).

## Data Availability

Data are unavailable due to privacy or ethical restrictions.
